# Supplementary figures and images for: A Four-Probiotic Regime to Reduce Surgical Site Infections in Multi-Trauma Patients
Source: Nutrients. 2022 Jun 24;14(13):2620. doi: 10.3390/nu14132620 (PMC9268677; doi:10.3390/nu14132620)

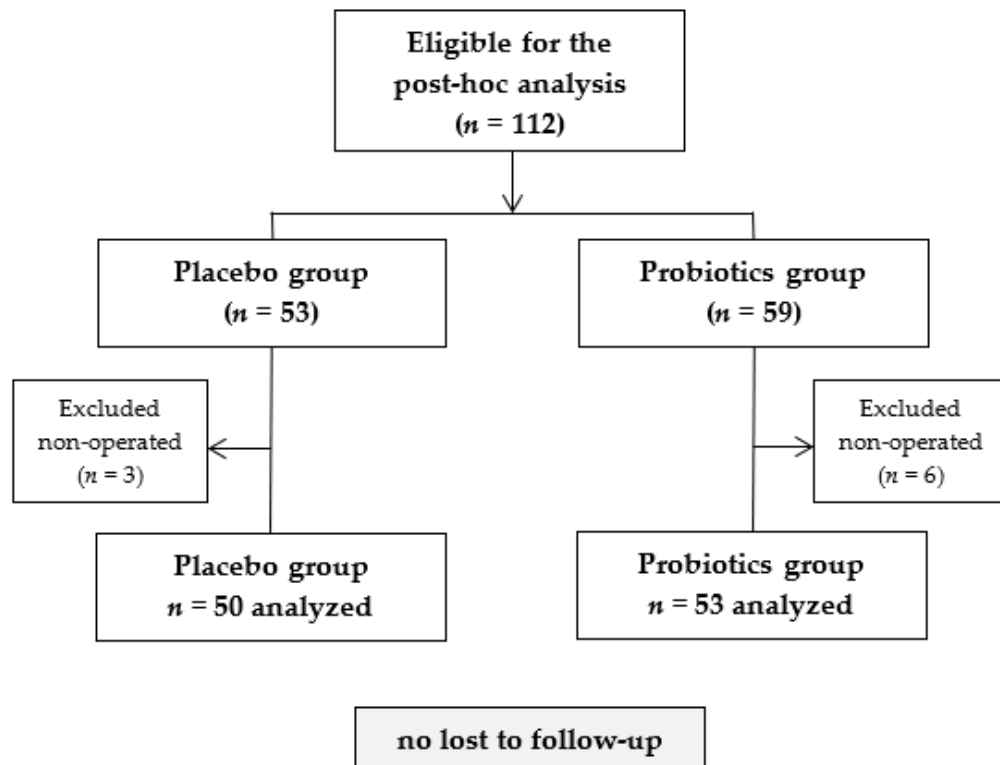

**Figure S1:** Study Flow Chart

Supplement: Supplementary file 1 [file nutrients-14-02620-s001.zip › nutrients-1775105-supplementary.pdf]
